# Microscopic feature of lymph node anthracosilicosis adherent with pulmonary artery: a case report

**DOI:** 10.1186/s13019-023-02348-5

**Published:** 2023-08-21

**Authors:** Junichi Murakami, Toshiki Tanaka, Yoshinobu Hoshii, Kimikazu Hamano

**Affiliations:** 1 Department of Surgery and Clinical Science, Division of Chest Surgery, Yamaguchi University Graduate School of Medicine, 1-1-1 Minami-Kogushi, Ube, 755-8505 Yamaguchi Japan; 2 Department of Diagnostic Pathology, Yamaguchi University Hospital, Ube, 755-8505 Yamaguchi Japan

**Keywords:** Lung cancer surgery, Anthracosilicosis, Lymph nodes, Dense adhesion, Pulmonary artery

## Abstract

**Background:**

Although thoracic surgeons occasionally encounter dense adhesions of interlobar lymph node anthracosilicosis with the pulmonary artery, adhesiolysis may be challenging. Besides, characteristic microscopic features of the adherent lesion remain limited.

**Case presentation:**

During a thoracoscopic right upper lobectomy of a patient with stage IA3 primary lung adenocarcinoma, several interlobar lymph nodes adhered to the posterior ascending branch of the pulmonary artery to the right upper lobe were noted. After an unplanned conversion to a thoracotomy to avoid massive bleeding, the pulmonary artery branch was safely isolated. Microscopic examination revealed lymph node anthracosilicosis proximal to the peripheral pulmonary artery wall, with granulomatous inflammation. The adventitial stroma of the pulmonary artery developed into dense and borderless fibrous tissue with dust-laden macrophages.

**Conclusions:**

Our pathological findings on lymph node anthracosilicosis provide substantial evidence that adhesions between lymph nodes and the pulmonary artery walls may develop into dense and borderless fibrous tissue. This finding would remind thoracic surgeons that adhesiolysis could cause injury to the pulmonary artery.

**Supplementary Information:**

The online version contains supplementary material available at 10.1186/s13019-023-02348-5.

## Background

Thoracic surgeons occasionally encounter dense adhesions of lobar or interlobar lymph nodes (LNs), with the pulmonary artery (PA), without malignant invasion [1, 2]. Forced adhesiolysis may be challenging for thoracic surgeons since it may cause life-threatening vascular injury. However, the characteristic microscopic features of these dense adhesions remain unclear. We report the histopathological findings of the adhesions between interlobar LNs and the PA.

## Case presentation

**Fig. 1:**
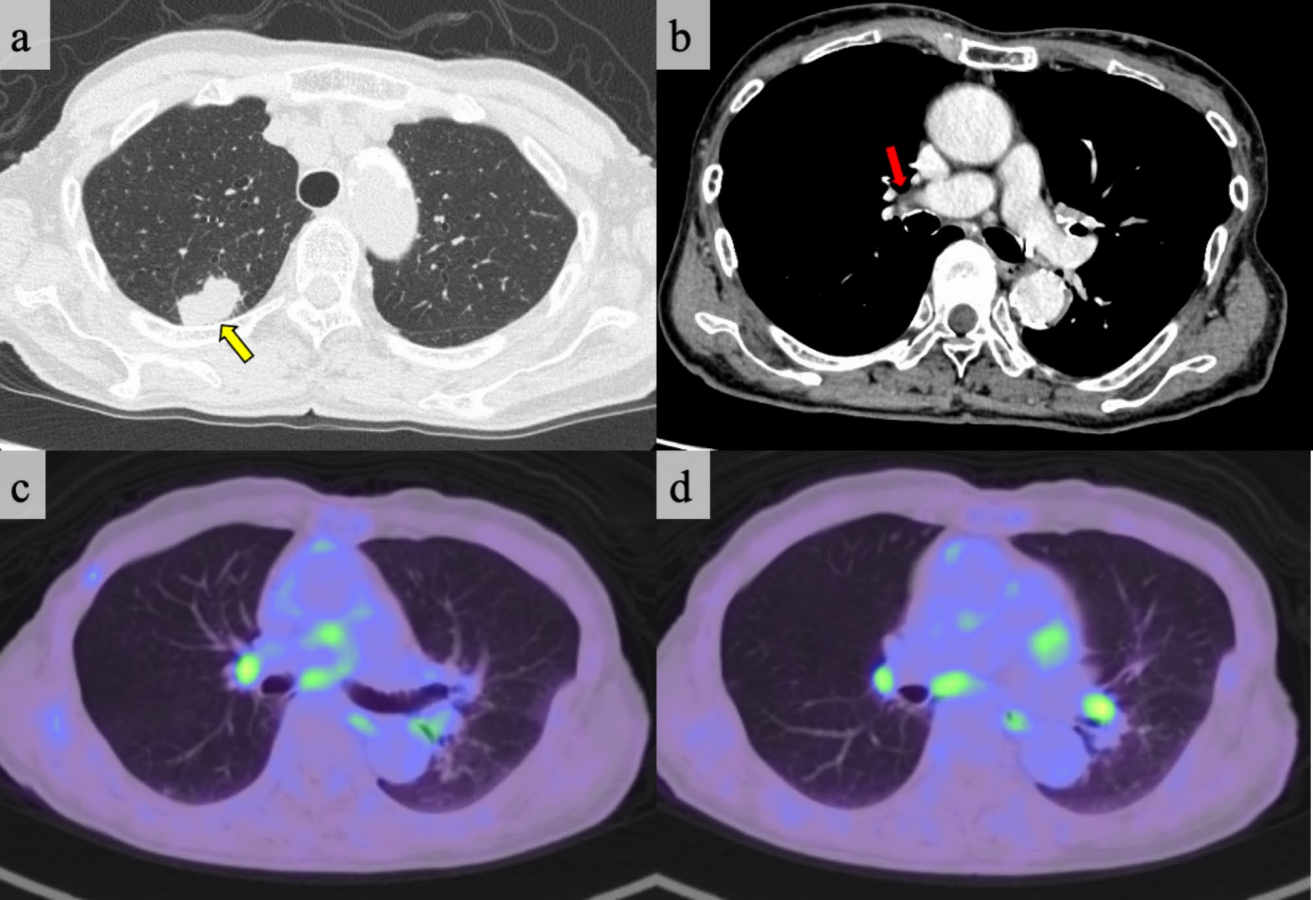
Preoperative chest computed tomography findings. The lung tumor is marked with a yellow arrow. (a) Hilar lymph nodes with neither enlargement nor calcification are marked with a red arrow. (b) Preoperative fluorodeoxyglucose-positron emission tomography images of the chest. Multiple hypermetabolic lymph nodes are visible at the bilateral hilar and mediastinal areas. c and d

A 91-year-old female, who has worked in the agriculture sector for over five years, presented to the outpatient clinic for further evaluation of a lung tumor that was incidentally noted. The patient had an ECOG-performance status of 0 and an unremarkable past medical history. No abnormalities were observed on routine blood examination and serum tumor marker analysis. Chest computed tomography (CT) revealed an approximately 2.5 cm-sized mass in the right upper lobe of the lung [Fig. 1A]. No enlargement or calcification of the hilar LNs was noted [Fig. 1B]. Fluorodeoxyglucose-positron emission tomography (FDG-PET) revealed high tumor uptake, with a maximal standardized uptake value (SUVmax) of 21.5, and diffusely-arranged bilateral hilar and mediastinal nodes, with SUVmax of 3–5, diagnosed as nonspecific FDG accumulation [Fig. 1C and D]. The tumor was identified to be an adenocarcinoma on a CT-guided biopsy. The patient was diagnosed with stage IA3 primary lung adenocarcinoma, according to the eighth edition of TNM classification of lung cancer.

**Fig. 2:**
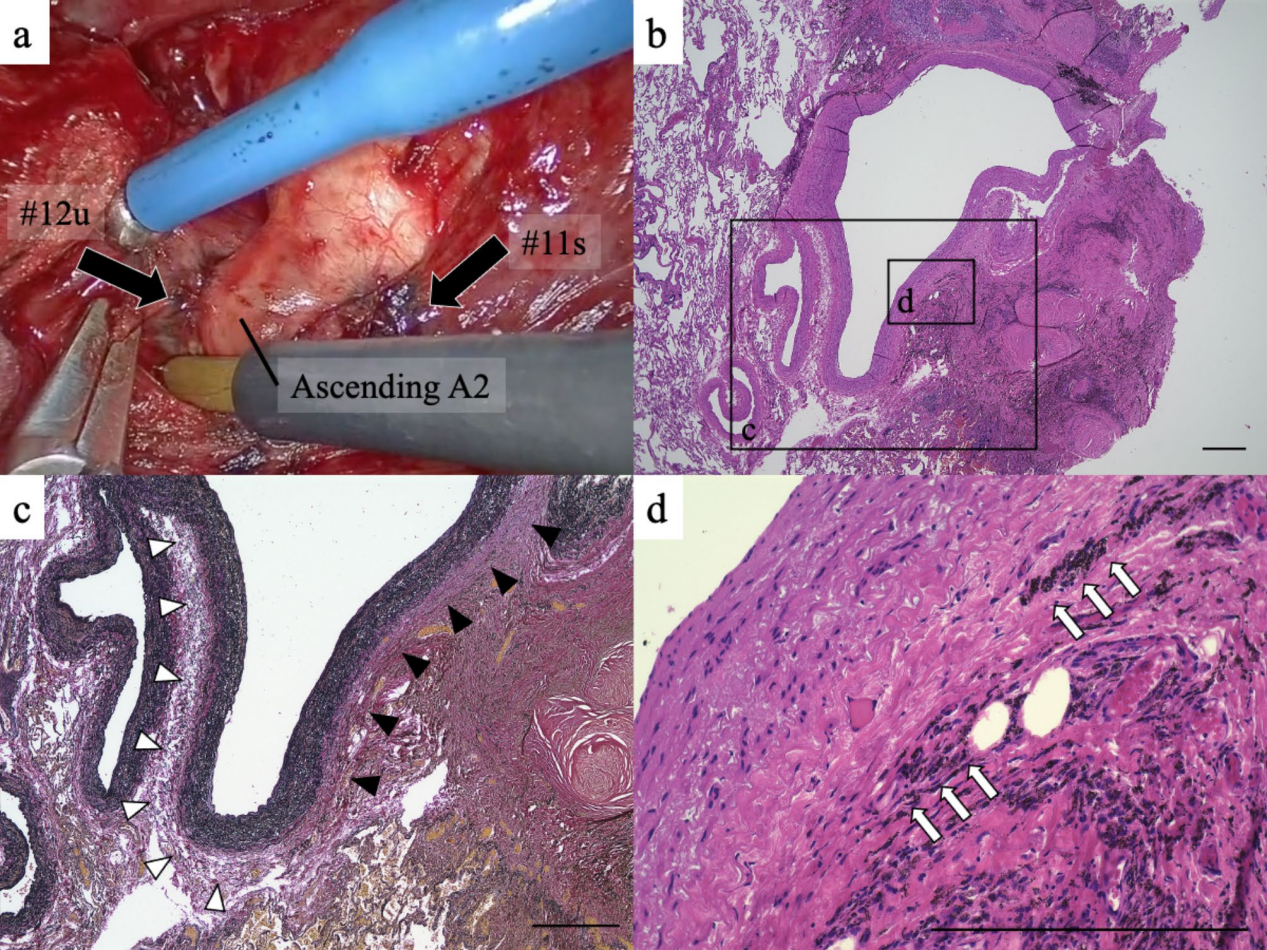
The lymph node (LN) anthracosilicosis and granulomatous inflammation of adventitia in the pulmonary artery (PA) are shown in microscopy. a. Intraoperative photograph. b, d, Hematoxylin–eosin staining. c, Elastica van Gieson staining. Black arrows: macroscopically infiltrating LN to PA. White arrowheads: normal adventitia. Black arrowheads: dense and borderless fibrous tissue. White arrows: dust-laden macrophages. The bars indicate 200 μm, respectively

Thoracoscopic right upper lobectomy, using the four-port method, was performed for the primary lung cancer. The upper lobar branch of the superior pulmonary vein and superior arterial trunk were isolated using an auto-stapler. Intraoperatively, multiple adherent LNs were observed around the upper bronchus and adjacent tissues. Several interlobar LNs (#12u and #11s) were strongly adherent with, and partially infiltrated to, the posterior ascending branch of the PA (ascending A2). Accurate dissection would have been challenging [Fig. 2A]. Intraoperative frozen section analysis confirmed the absence of metastases in one of the hilar LNs (#12u). We decided to convert from a thoracoscopic approach to thoracotomy in preparation for potential PA injury because the upper lobar branch of the superior pulmonary vein was already transected. After performing a 12 cm skin incision, the posterior ascending branch of the PA was safely isolated via proximal double ligation, with transfixing sutures and a distal single adherent LN-transfixing suture, while retaining a part of the hilar LNs. The upper lobe bronchus was secured using an auto-stapler. Only the hilar LNs were dissected. The mediastinal LNs were left untouched. The total operative time was 307 min, and the estimated blood loss was 80 mL.

The tumor was histopathologically diagnosed as a well-differentiated apT1cN0M0 stage IA3 adenocarcinoma. Microscopic examination revealed anthracosilicosis in the lobar LNs, which presented as fibrotic nodules in a concentric onion-skinned arrangement of hyalinized collagen bundles, in extensive proximity to the peripheral PA wall, accompanied by granulomatous inflammation [Fig. 2B]. By contrast, fibrotic nodules were inconspicuous in the lung parenchyma. The LNs with silicotic changes had no lymphoid lobules surrounded by lymph-filled sinuses, tumor metastasis, and calcifications. The adventitial stroma of the PA, which typically consists of a loose extracellular matrix scaffold where a boundary between LN anthracosilicosis and PA is dissected [Fig. 2C], developed into a dense and borderless fibrous tissue with variable dust-laden macrophages [Fig. 2D]. No cancerous tissue was observed at the PA stump.

The patient’s postoperative course was uneventful, and no further complications were noted. The chest tube was removed one day postoperative after confirming that there was no air leakage. The patient was discharged on the seventh postoperative day. The patient was regularly followed up on an outpatient basis, with routine blood examinations and CT scans every three months. No recurrence was observed two years later.

## Discussion and conclusions

Anthracosilicosis is a fibrotic lung disease caused by long-term inhalation of silica, dust-containing amorphous silica, or carbonaceous particles, in the occupational environment, such as construction, mining, quarrying, tunneling, jewelry, and agriculture [3]. It is an occupational disease closely associated with one’s occupational history. Phagocytosis of silica or carbonaceous particles in the distal airway triggers an inflammatory cascade, with subsequent fibrosis in the hilar LNs and lung parenchyma [4]. The silica- or carbonaceous particles-induced fibrosis and inflammation are associated with various pathological findings such as fibrotic nodules, anthracotic pigment, and calcifications [3]. In our case, occupational exposure to respirable crystalline silica and carbonaceous particles in agricultural work may have developed a borderless fibrous tissue between the PA, via an inflammatory process activated by the phagocytosis of macrophages in the lobar LNs.

In practice, performing a wedge lung resection in response to PA-adherent LN may be the best option. We decided to convert to a thoracostomy to avoid massive bleeding because all the superior vessels, except A2, had already been divided. Regarding surgical strategies to isolate PA adhesions with non-metastatic LN, the En-masse technique, which involves a simultaneous dividing of the pulmonary vessels and bronchi at the hilum, and transfixing suture along with adherent LN, may be appropriate [2, 5].

Lymph node-related issues have been reported to be the leading cause of unplanned open thoracotomies during thoracoscopic lung resection [6, 7]. This may be due to the difficulty of preoperative radiological prediction in adhesions between LNs and the PA. Minimum intensity projection CT has been reported as a promising method to preoperatively assess the degree of LN adhesion to adjacent structures [8]. Still, this objective evaluation method is somewhat complicated and esoteric. Although a preoperative bronchoscopic assessment was not performed in this case, Takeda et al. reported that dark bronchial pigmentation, the size of hilar LNs, and their combination were useful for predicting PA-adherent LNs [9]. Further, it may be challenging to distinguish inflammatory infiltrates from invasive metastases to the LNs. Although preoperative FDG-PET is a noninvasive procedure widely performed for diagnosing LN involvement, nodal staging with FDG-PET is insufficient due to the high false-positive rates brought about by silicosis [10]. Therefore, intraoperative frozen section biopsy for adherent LNs is recommended to exclude the possibility of metastasis, as in our case. However, intraoperative biopsy cannot completely rule out the presence of micrometastasis in LNs invading the PA. LNs that are hard and with anthracotic pigment infiltrating the vessel wall are often due to inflammation rather than tumor involvement. In the future, intraoperative imaging using ultrasound and augmented reality technology, such as artificial intelligence, may help identify areas of dense adhesions and exclude the possibility of LN metastasis.

There are few opportunities to examine the pathological features of the boundary between LN anthracosilicosis and the PA in detail because thoracic surgeons frequently dissect the adherent PA with LN anthracosilicosis, while leaving a part of the LN, after confirming the absence of LN metastases [2, 11]. Therefore, there are no intraoperative reports on the histopathological findings of a dense adhesion between LN anthracosilicosis and the PA. Our pathological findings suggest that it was too delicate to dissect the adventitia of the PA in contact with LN anthracosilicosis using common maneuvers. The microscopic details of this case can help gain a better understanding of the technical difficulty in forced adhesiolysis and contribute to increased safety when addressing the problem, despite the limitation of this being only a single case.

Our pathological findings prove that a boundary between the anthracosilicosis LN and PA may develop into an undissectable dense and borderless fibrous tissue, with granulomatous inflammation and that forced adhesiolysis against this boundary could cause PA injury.

### Electronic supplementary material

Below is the link to the electronic supplementary material.

**Additional File 1:** Video file

## Data Availability

The data is available and can be sought with the authors on request.

## Abbreviations

***LNs***: Lymph nodes
***PA***: Pulmonary artery
***CT***: Computed tomography
***FDG-PET***: Fluorodeoxyglucose-positron emission tomography
***SUVmax***: Maximal standardized uptake value
